# Integrating Network Pharmacology and *In Vivo* Model to Investigate the Mechanism of Biheimaer in the Treatment of Functional Dyspepsia

**DOI:** 10.1155/2022/8773527

**Published:** 2022-05-27

**Authors:** Chun Wang, Nuermanati Huanbieke, Xiaoxia Cai, Shuyan Gao, Tianfang Du, Ziqian Zhou, Zulipikaer Wusiman, Malikam Matturzi, Silafu Aibai, Zhi-Jian Li

**Affiliations:** ^1^College of Pharmacy, Xinjiang Medical University, Urumqi 830011, China; ^2^Xinjiang Institute of Traditional Uyghur Medicine, Urumqi 830049, China; ^3^Uygur Medical Hospital of Xinjiang Uygur Autonomous Region, Urumqi 830049, China

## Abstract

**Objective:**

Biheimaer (BHM) is a hospital formulation for clinical treatment of dyspepsia and acid reflux, based on Compatibility Theory of Traditional Chinese Medicine. This study anticipated to elucidate the molecular mechanism of BHM against Functional dyspepsia via combined network pharmacology prediction with experimental verification.

**Methods:**

Based on network pharmacology, the potential active components and targets of BHM in the treatment of functional dyspepsia were explored by prediction and molecular docking technology. The results of protein–protein interaction analysis, functional annotation, and pathway enrichment analysis further refined the main targets and pathways. The molecular mechanism of BHM improving functional dyspepsia mice induced by L-arginine + atropine was verified on the basis of network pharmacology.

**Results:**

In this study, 183 effective compounds were screened from BHM; moreover, 1007 compound-related predicted targets and 156 functional dyspepsia-related targets were found. The results of enrichment analysis and *in vivo* experiments showed that BHM could regulate intestinal smooth muscle contraction to play a therapeutic role in functional dyspepsia by reducing the expression of NOS3, SERT, TRPV1, and inhibiting the inflammatory cytokine (IL-1*β*, TNF-*α*) to intervene the inflammatory response in mice.

**Conclusions:**

This study revealed the molecular biological mechanisms of the Traditional Chinese Medicine formulation of BHM in functional dyspepsia by network pharmacology and experimental verification, meanwhile provided scientific support for subsequent clinical medication.

## 1. Introduction

Functional dyspepsia is one of the most common gastrointestinal diseases. Studies have shown that the prevalence of functional dyspepsia in Western populations is 9.8% to 40%, and the Eastern population is lower at 5.3% to 28%. [1, 2]. The incidence of the disease is gradually increasing, and the pathogenesis has not been fully elucidated. At present, most modern medical treatments use *Helicobacter pylori* eradication therapy, proton pump inhibitors, histamine-2 receptor antagonists, central neuromodulators, prokinetics, etc [3]. In clinical application, the above-mentioned drug treatment has modest effectiveness and none of the treatments has been proven to change the long-term natural course of functional dyspepsia, and there are problems with high treatment costs and adverse effects caused by long-term application [4, 5]. Herbal medicines, such as traditional Chinese medicine compound preparation, are expected to become effective and safe therapy options for functional dyspepsia treatment.

Biheimaer (BHM) is a hospital formulation for clinical treatment of dyspepsia and acid reflux, based on Compatibility Theory of Traditional Chinese Medicine, composed of *Artemisia rupestris* (L.), *Mentha longifolia* (L.), *Cuminum cyminum* (L.), dried ginger, *Elettaria cardamomum* (L.) Maton, pomegranate seed, and other components, clearing away heat and toxic materials, regulating gastrointestinal function, reducing hiccups, and belching. Our group's previous studies have confirmed that *Artemisia rupestris* (L.), as a principal drug, has anti-inflammatory effects, improves immune function, promotes gastrointestinal digestion, and reduces reflux esophagitis and functional dyspepsia symptoms [6]. The effect of *Mentha longifolia* (L.) on reducing food stagnation and flatulence has been confirmed by clinical studies, which reveals that Peppermint Oil in *Mentha longifolia* (L.) has a dose-related effect on gastrointestinal motility [7]. *Cuminum cyminum* (L.) has the effect of treating indigestion and abdominal pain, which is recorded in the famous traditional Chinese medicine monograph “Puji Fang.” The role of dried ginger in resisting *Helicobacter pylori* has been widely reported [8, 9]. Elettaria cardamomum (L.) Maton and pomegranate seed are widely used as traditional medicine or food to regulate gastrointestinal function all over the world [10–12]. However, the composition of BHM is complex and its pharmacological mechanism of action has not yet been clarified.

**Figure 1:**
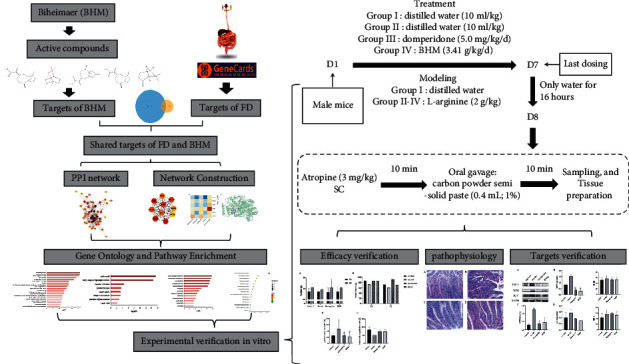
The flowchart for dissecting the mechanisms of action of BHM in the treatment of functional dyspepsia.

Network pharmacology was proposed by Hopkins in 2007. With the help of network analysis, the relationship between drugs and disease targets is studied through “multi-component, multi-target, and multi-pathway.” Network pharmacology believes that the essence of disease is genes or encoded proteins, represented by one or more nodes in the network, are in an unbalanced state, which coincides with the law of occurrence and development of disease in the theory of Chinese medicine. Traditional Chinese Medicine believes that the essence of the disease is the deviation of the yin and yang attributes of the human body, and the deviation is corrected through multiple angles and multiple channels of traditional Chinese medicine compounds. In this study, network pharmacology was used to predict key targets that have a critical function in BHM treatment of functional dyspepsia, and pathway enrichment was used to predict the critical mechanisms. Ultimately, these mechanisms were verified through molecular docking and *in vivo* experiments [13]. A brief flowchart of the method is shown in Figure 1.

## 2. Materials and Methods

### 2.1. Collection of Active Compounds and Putative Targets

The compounds in BHM were identified from literature search and Traditional Chinese Medicine Systems Pharmacology Database and Analysis Platform (TCMSP) (https://old.tcmsp-e.com/tcmsp.php). When herbs were listed in TCMSP, active compounds were screened with thresholds value of oral bioavailability (OB) ≥ 30% and drug similarity (DL) ≥ 0.18 [14]. The compounds contained in the rest of the herbs were collected by consulting the literature and performing drug-like screening based on Lipinski in Swiss ADME (https://www.swissadme.ch/).

Potential targets associated with candidate compounds are predicted in Swiss Target Prediction System [15] (https://www.swisstargetprediction.ch/). The identified targets of functional dyspepsia were collected from Gene Cards database (https://www.genecards.org/); the targets were searched by making “Functional dyspepsia” as key word. In order to understand the relationship between disease targets and BHM-related targets more intuitively, two sets of targets were input into Venny 2.1 (https://bioinfogp.cnb.csic.es/tools/venny/), respectively, to obtain the intersection targets.

### 2.2. Construction of the Protein–Protein Interaction (PPI) Network

The above intersection target proteins were imported into String platform (https://string-db.org/) to explore the relations of protein–protein interaction (PPI) [16], and then input into Cytoscape 3.7.0 to obtain the PPI network. The key targets obtained from the PPI network were input into Metascape (https://metascape.org/) for the enrichment analysis of Gene Ontology (GO) and Kyoto Encyclopedia of Genes and Genomics (KEGG) pathways. The “Input as species” and “Analysis as species” set as “H. sapiens.” Pathways satisfying *P* < 0.05 were considered as significant.

### 2.3. Molecular Docking Simulation

The molecular structures of the ligand and the target protein were downloaded from The Protein Data Bank (PDB) (https://www.rcsb.org/pdb/) and PubChem database (https://pubchem.ncbi.nlm.nih.gov/), and waters and ligands were removed by PyMOL. The protein structure was hydrogenated by imported into AutoDock 1.5.6. Finally, docking the processed protein and active compound in AutoDock to verify the molecular binding ability and calculate the minimum binding energy. The docking results were visualized using PyMOL.

### 2.4. Experimental Verification In Vivo

#### 2.4.1. Chemicals and Reagents

Biheimaer (BHM, Xinjiang Uygur Pharmaceutical Co., LTD., China; All herbs are comminuted and sifted through 100 mesh sieve); L-arginine (Shanghai Yuanye Biotech, China), atropine (Henan Runhong Pharma, China), mosapride (Hansoh Pharma, China), IL-1*β* and TNF-*α* ELISA kits (Multi Sciences, China); Polyclonal antibodies *β*-actin, SERT, NOS3 (Bioss, China), TRPV1 (ABclonal, China), electrogenerated chemiluminescence (ECL) and color liquid (biosharp, China).

#### 2.4.2. Establishment of Mouse Functional Dyspepsia Model and Animal Administration

Male Kunming (KM) mice (*n* = 40), weight 18–22 g, were obtained from the Animal Experiment Center of Xinjiang Medical University. The animals were bred in the animal room of the Xinjiang Institute of Traditional Uygur Medicine with the room temperature of 20–26°C and the relative humidity of 40–70%. Before the experiment, all animals were adapted to rearing for 3 days and quarantined. Animals with abnormal growth were excluded and randomly divided into groups. The protocols for the experiments were approved by the Xinjiang Institute of Traditional Uygur Medicine Ethics Committee on animal experimentation (Approval code: nGLP-YZH-12-005) and conducted in accordance with internationally accepted principles for the use and the care of laboratory animals.

40 healthy male mice were randomly divided into 4 groups: control group (group I), model group (group II), Mosapride group (group III), and BHM group (group IV). The positive drug mosapride (5.0 mg/kg/d) (group III), and the BHM (3.41 g/kg/d) (group IV) is given daily by oral gavage for 7 days. Except for the control group, the other groups were given L-arginine (2 g/kg) orally for 7 consecutive days. Before sacrifice, all of the mice were fasted without water restriction for 16 hours. On the 8th day, 10 minutes after atropine was given (3 mg/kg) by subcutaneous injection (SC), carbon powder semi-solid paste (0.4 mL; 1%) was fed into the stomach, and sacrificed after another 10 minutes.

#### 2.4.3. Gastrointestinal Motility as Indicated by Carbon Indicator

After the mice were sacrificed, the stomach and intestine were dissected. After weighing the total weight of the stomach, cut the stomach body along the greater curvature of the stomach to obtain the net weight of the stomach. The gastric content is calculated by the total weight of the stomach minus the net weight. At the same time, the small intestine was quickly cut from the pylorus to the ileocecal area. After gently peeling the mesentery, the overall length between the pylorus and the ileocecal area and the distance from the pylorus to the front of the carbon paste were measured. Intestine was fixed in 4% PFA fix solution at room temperature for 24 h. The rate of gastric residual and intestinal propulsion rate are calculated by this formula:

(1) $$\begin{array}{c} \text{gastric residual rate}\left(\%\right)=\frac{W_{\left(\text{gastric content}\right)}}{0.56_{\left(\text{carbon paste}\right)}}\times 100\%,\\ \text{intestinal propulsion rate}\left(\%\right)=\frac{L_{\text{propulsion distance}}}{L_{\left(\text{total intestine}\right)}}\times 100\%. \end{array}$$

#### 2.4.4. Biochemical Assays

The cytokines levels of IL-1*β* and TNF-*α* were quantified by ELISA kits following manufacture instruction separately, and measured OD values by Spark microplate reader (Tecan, Switzerland), and the corresponding concentrations were calculated.

#### 2.4.5. Histomorphology Observation

The duodenum tissues were stained by hematoxylin-eosin (HE) with paraffin section method. The pathological change was estimated by a third-party pathologist who did not participate in the experiment (magnification, 200x).

#### 2.4.6. Western Blot Analysis

50 mg of the duodenum tissue of mice were extracted protein with 300 *μ*L RIPA buffer, and determined by BCA assay. The protein samples were separated by 8% SDS-polyacrylamide gel. The PVDF membranes were blocked with 5% skim milk for 2 h, and primary antibodies including TRPV1, SERT, eNOS (NOS3), and *β*-actin were added overnight (4°C). The membranes were incubated with the secondary antibody for 1 h and visualized using an ECL developer and multifunctional imaging system (Vilber Fusion Fx6, France). The gray value of the target bands was analyzed using Image J, and the relative expression of proteins was expressed as target protein/*β*-actin.

#### 2.4.7. Statistical Analysis

All values are expressed as mean ± standard deviation. SPSS software (version 20.0) was used for the statistical analysis. One-way Analysis of Variance (ANOVA) was used for the comparison between groups. *P* < 0.05 was considered significant in statistics.

## 3. Result

### 3.1. Results of Screening the Targets of BHM and Functional Dyspepsia

**Figure 2:**
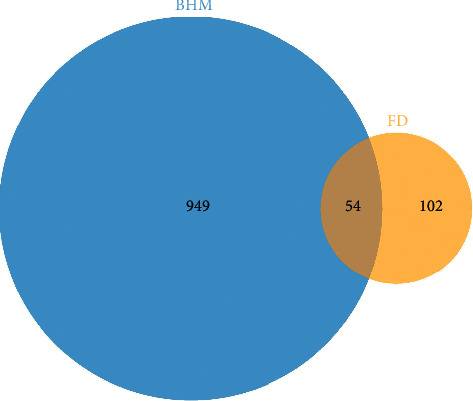
Venn map of compound BHM target for improving functional dyspepsia.

Through screening, 183 active components were identified, and verified chemical structures subsequently from the PubChem database. Based on that, 1007 compound-related targets were obtained after eliminating the duplicates. Moreover, 156 functional dyspepsia-related targets were confirmed from the GeneCards database. Depending on the 1007 putative targets of the BHM and the 156 functional dyspepsia-related targets, 54 integrated targets were identified as potential therapeutic targets of BHM against functional dyspepsia (Table 1). A Venn diagram was established to clarify the relationship between BHM and functional dyspepsia-related targets (Figure 2).

**Figure 3:**
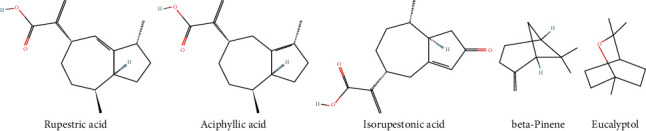
Chemical structures of the most relevant active components.

By comparing the information in compounds and targets, it was found that rupestric acid, aciphyllic acid, isorupestonic acid, beta-pinene, and eucalyptol in BHM should be the most relevant active component related to functional dyspepsia, and the above 5 components were selected for the following efficacy verification (Figure 3).

### 3.2. Construction of PPI Network

**Figure 4:**
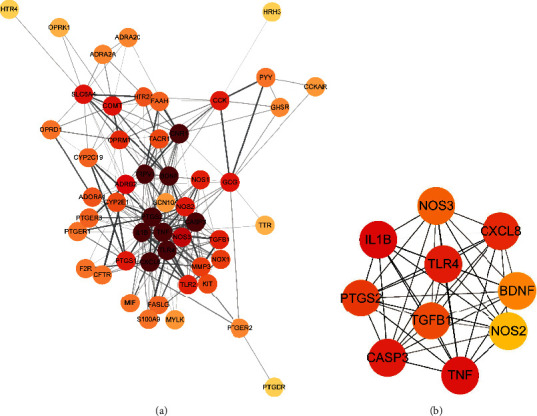
Results of PPI network analysis of targets of BHM for improving functional dyspepsia. (a) PPI network; (b) core targets.

54 integrated targets were uploaded to the String database to determine interaction relationship. Then, the protein isolated outside the network was eliminated and the protein interactions (confidence level: 0.4) were imported into Cytoscape v3.8.0 to PPI networks, which consisted of 51 nodes and 258 edges, as shown in Figure 4(a). CytoHubba, the plug-in of Cytoscape, sorted all genes and acquired 10 core targets, which is widely used to predict key targets or sub-networks (Figure 4(b)).

### 3.3. Enrichment Analysis of Related Pathways and Biological Process

**Figure 5:**
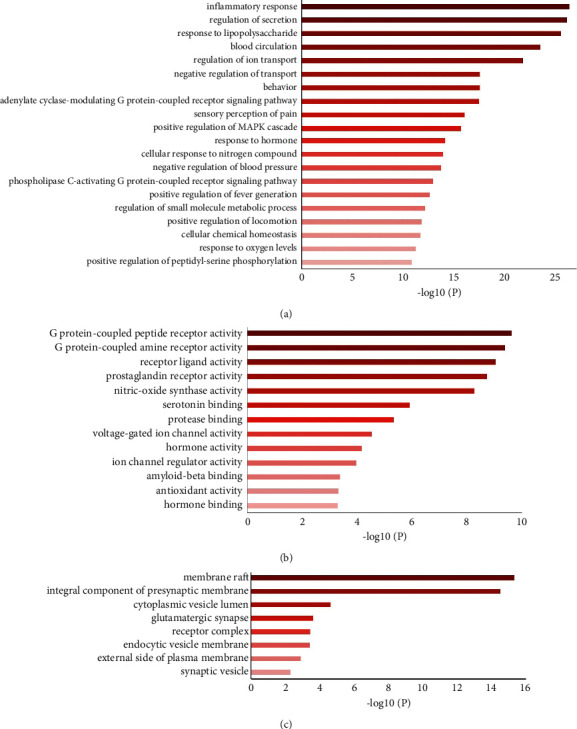
GO functional enrichment. (a) Top 20 biological processes of GO. (b) Top 13 molecular functions of GO. (c) Top 8 cellular components of GO.

**Figure 6:**
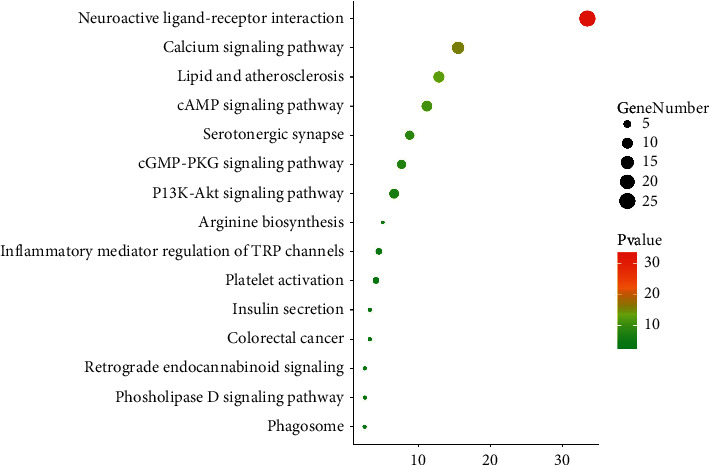
KEGG pathway analysis of top 15 enriched. The importance of pathways is evaluated and ranked by gene number and a -Log10 (*P* value) bar chart.

The key targets obtained above were input into Metascape for annotation analysis. Here, top20, top13, and top 8 in biological processes (BP), molecular function (MF), cellular component (CC), are shown in Figure 5, respectively. In addition, the top 20 KEGG pathways satisfying *P* < 0.05 were visualized by dot plot (Figure 6), including the Neuroactive ligand-receptor interaction, Calcium signaling pathway, Serotonergic synapse, and Inflammatory mediator regulation of TRP channels.

### 3.4. Molecular Docking Validation

**Figure 7:**
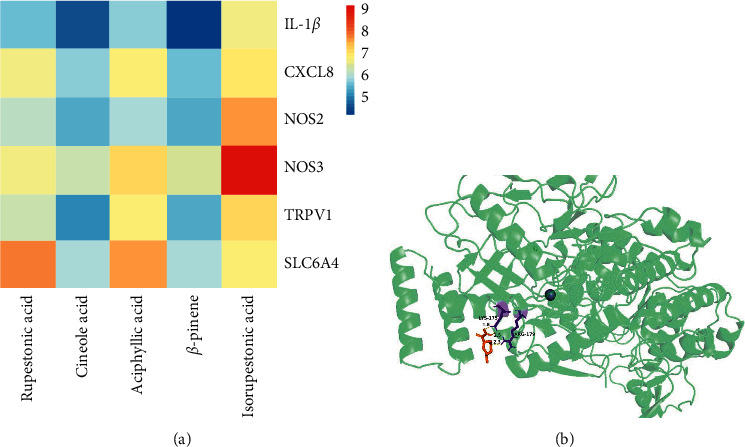
(a) The heat map of binding energy between the active compounds and IL-1*β*, CXCL8, NOS2, NOS3, TRPV1, and SLC6A4. (b) Molecular docking site of NOS3 and Isorupestonic acid.

The minimal binding between bioactive components (rupestric acid, aciphyllic acid, isorupestonic acid, and beta-pinene, eucalyptol) and core proteins (IL-1*β*, CXCL8, NOS2, NOS3, TRPV1, and SLC6A4) was simulated and analyzed by molecular docking software (Figure 7(a)). According to literature reports, binding energy of less than -5.0 kcal/mol is the standard for stable binding of ligands and receptors during molecular docking, and the lower binding energy means the molecular structure is more stable [17]. Isorupestonic acid has the maximum binding energy with NOS3 (−9.10 kcal/mol), which may play a significant role in the effect of isorupestonic acid on functional dyspepsia. The docking site of isorupestonic acid and NOS3 is visualized in Figure 7(b).

### 3.5. BHM Improved Gastrointestinal Function on Functional Dyspepsia Model Mice

**Figure 8:**
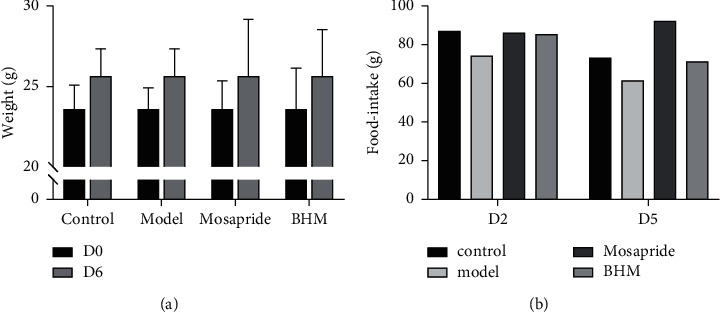
Effects of 7-day treatment on weight and food intake in mice. (a) Average weight of each group. (b) Change of food intake.

Mice in the control group maintained a stable increasing weight during the experimental process, while the functional dyspepsia model group mice gained more slowly due to the inhibition of gastrointestinal function (Figure 8(a)). In addition, the food intake of the mice in model group was lower than the control group since the modeling. Compared to the model group, the food intake and body weight of the mice in the Mosapride and BHM groups were higher, and the increase in the food intake of Mosapride was more significant (Figure 8(b)). It indicated that Mosapride and BHM promoted the recovery of digestive function.

### 3.6. Effect on Gastric Emptying and Intestinal Propulsion

**Figure 9:**
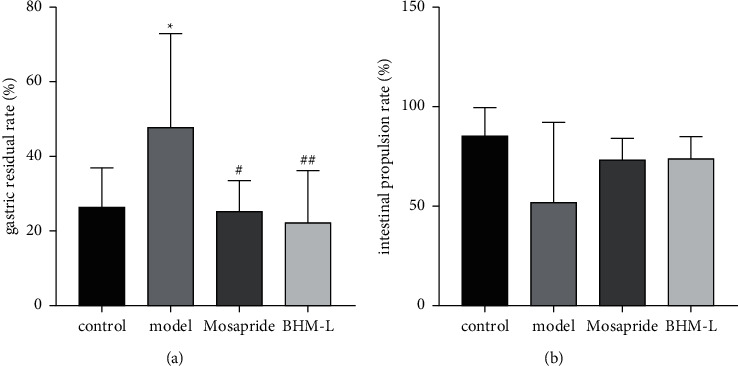
Effect of BHM on gastric emptying and intestinal propulsion in mice. (a) Calculated gastric residual rate. (b) Calculated intestinal propulsion rate. ^*∗*^*P* < 0.05*vs*. the control; ^#^*P* < 0.05*vs*. the model; ^##^*P* < 0.01*vs*. the model.

As shown in Figure 9, after treatment with mosapride and BHM, the gastric residual rate of functional dyspepsia model mice was significantly reduced and intestinal advancement rate also increased, indicating that BHM can improve gastric emptying in mice, which was consistent with the positive drug group.

### 3.7. Result of Histomorphology

**Figure 10:**
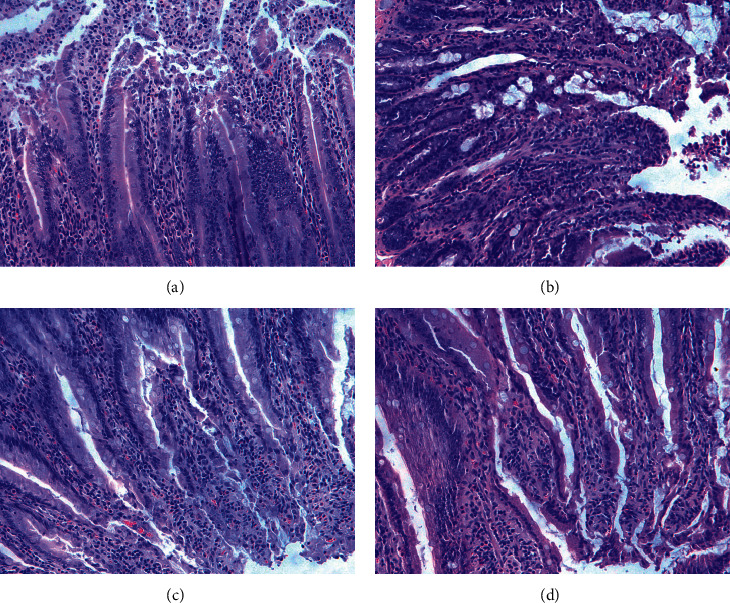
Effects of BHM on histomorphological changes in the duodenum of mice (200x). (a) Control group. (b) Model group. (c) Mosapride group. (d) BHM group.

In comparison to control group, there were more infiltrated inflammatory cells and denatured necrotic mucosa in the duodenum of mice in model group; in contrast to model group, the infiltration of inflammatory cells and the degree of degeneration and necrosis of mucous epithelium were improved in the duodenum of mice in Mosapride and BHM groups (Figure 10).

### 3.8. Effects of BHM on IL-1*β* and TNF-*α* Levels

**Figure 11:**
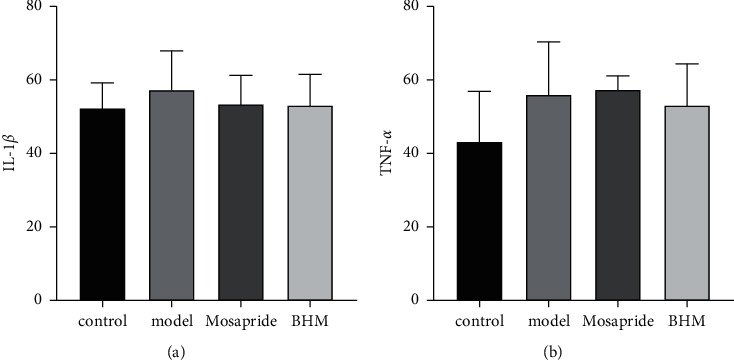
Content of IL-1*β* (a) and TNF-*α* (b) in mice duodenum.

ELISA results (Figure 11) showed that IL-1*β* and TNF-*α* in the duodenum of mice in the model group were increased and decreased after administration, albeit not significantly (*P* > 0.05).

### 3.9. Expression of TRPV1, SERT, and NOS3 Proteins

**Figure 12:**
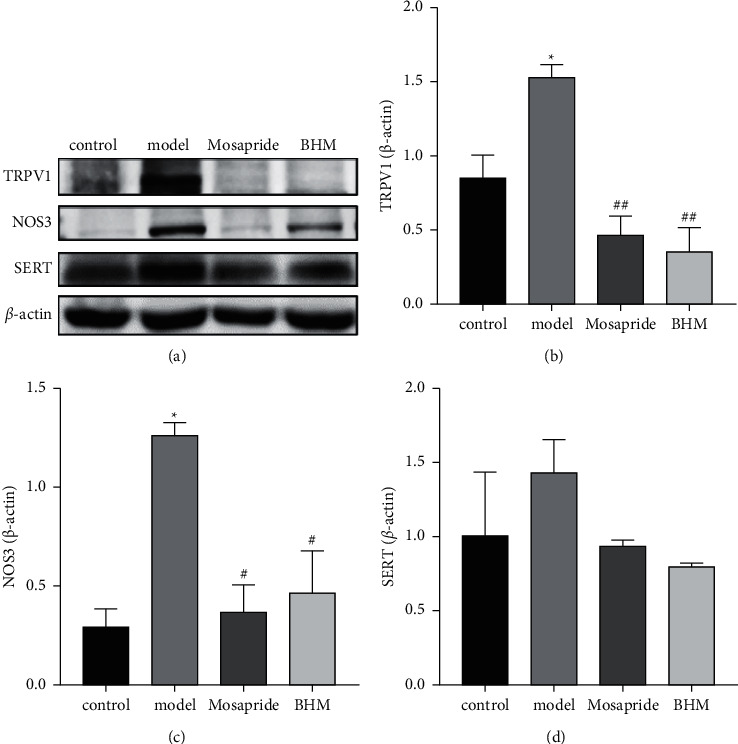
Effects of BHM on the expression of TRPV1, NOS3, and SERT proteins in the duodenum tissue (a). The expressions of TRPV1 (b), NOS3 (c), and SERT (d) were quantified by densitometry using ImageJ software and normalized with *β*-actin. ^*∗*^*P* < 0.05*vs*. the control; ^#^*P* < 0.05*vs*. the model; ^##^*P* < 0.01*vs*. the model.

As shown in Figure 12, the expression of TRPV1, NOS3, and SERT in the duodenum tissue of mice in model group was significantly higher than that in control group (*P* < 0.05), and the expression of TRPV1, NOS3, and SERT in the duodenum tissue of mice in BHM group was significantly lower than that in model group (*P* < 0.05). The results of *in vivo* experiments were consistent with the conclusions of network pharmacology.

## 4. Discussion

The current research road of network pharmacology is to establish a target prediction network model of specific disease and its prevention or treatment drug based on public and published data. The model predicts the target of the drug being studied and thereby constructs a drug-target-disease network to explore the potential pharmacological mechanism. Ultimately, the potential mechanism is verified by corresponding experiments such as molecular docking, *in vivo* or *in vitro* experiments [18]. BHM contains a variety of active components that can improve the digestive system functions through multiple targets, biological processes, and related pathways. The compound has a long history of clinical application, but few experiments involve the mechanism of action of BHM in the process of anti-functional dyspepsia. Therefore, this study investigated the potential mechanism and verified the exact molecular function of BHM in the treatment of functional dyspepsia.

In the present study, 1007 potential targets of the 183 components in BHM and 156 related targets of functional dyspepsia were identified. By comparing the information of quantity, contribution degree, and function of compounds and targets, the most potent compound is rupestric acid, aciphyllic acid, isorupestonic acid, beta-pinene, and eucalyptol. Relevant pharmacological studies in recent years have shown that they may have the effect of improving functional dyspepsia symptoms, involving gastroprotective, anti-inflammatory, and analgesic, promoting gastrointestinal motility [19, 20]. Construction of PPI network reviewed 54 related targets between BHM and functional dyspepsia. 10 core targets were further screened based on degree value. However, these bioinformatics data can only reflect drug-target-disease correlations. Causal relationships, such as up-regulation and down-regulation of the prediction mechanism, still need to be verified by appropriate experiments.

Among the overlapping targets between BHM and functional dyspepsia, IL1B, TNF, PTGS2, BDNF, TLR4, CXCL8, CASP3, CNR1, TRPV1, and NOS3 showed the highest degree values (top10) suggesting that they may be the main target of BHM for functional dyspepsia. These 10 target genes are divided into five directions related to inflammatory response, regulation of nitric-oxide synthase activity, neuroactive ligand-receptor interaction, and regulation of secretion according to their functions. This also reflects the characteristics of BHM's comprehensive regulation and coordinated restoration of body balance. With the largest degree values, IL1B and TNF were regarded as the more dominant of the overlapping targets. IL1B and TNF are important potent pro-inflammatory cytokines that play a key role in inflammatory response [21]. These pro-inflammatory cytokines are regulated by TLR4 through the TLR2/4-NFKB signaling pathway together with CXCL8 [22]. Low-grade inflammation of the duodenum is considered one of the main pathological mechanisms of functional dyspepsia. Several studies have now reported that systemic markers of inflammation were elevated and linked with both duodenal hyperpermeability and gastric emptying as well as dyspeptic symptoms [23, 24]. Other targets, such as TRPV1, is a non-selective cation channel to mediate proton influx, involved in various biological processes, including regulation of mediation of inflammatory pain and hyperalgesia [25]. Evidence suggests that up-regulating TRPV1 may contribute to visceral hypersensitivity, which occurs through the sensitization of afferent nerve fibers in the gut that constituted a mechanism for gastric hyper‐sensitivity in functional dyspepsia [26, 27]. Endothelial nitric oxide synthase (eNOS/NOS3) is one of the main pathways to produce NO that relaxes GI smooth muscle to regulate physiologic peristalsis [28]. L-arginine excess intake will up-regulate eNOS expression inducing NO concentration increase, which may be an important molecular mechanism underlying functional dyspepsia [29–31]. The increased IL1B, TNFT, RPV1, and NOS3 expression in the duodenum of functional dyspepsia model mice were significantly affected following BHM treatment, suggesting that BHM regulates duodenum functionality and reduces inflammatory response by targeting these genes.

The biological process of GO enrichment analysis was significantly enriched in inflammatory response, regulation of secretion, sensory perception of pain, and regulation of ion transport. The neuroimmune regulation, especially the NO synthase, in functional dyspepsia participates in the regulation of secretion and regulation of ion transport, which play important roles in the pathogenesis of functional dyspepsia [32–34]. NO synthase affects various cell functions, including calcium ion regulation, inflammatory cascades, neurotransmitter release, and signal transmission [35]. In this study, BHM significantly promoted gastrointestinal peristalsis, maintained the intestinal smooth muscle at normal contractile function, and prevented the intestinal muscle relaxation and gastroparesis of mice induced by L-arginine.

KEGG pathway annotation analysis obtained a number of related clustering pathways, which mainly include the neuroactive ligand-receptor interaction, calcium signaling pathway, serotonergic synapse, and inflammatory mediator regulation of TRP channels. This suggests that BHM can comprehensively regulate multiple signaling processes relevant to the pathophysiology of functional dyspepsia. The neuroactive ligand-receptor interaction pathway is composed of cell membrane receptors, such as capsaicin receptor, adrenergic receptor, muscarinic acetylcholine receptor, and 5-hydroxytryptamine receptor, which are involved in the transduction of intracellular and extracellular signals [36–38]. In addition, the calcium signaling pathway is connected to synaptic signaling and the nitrergic synthesis pathway is activated by the opening of voltage-gated calcium channels and ultimately triggers the release of NO [39], and a fundamental mechanism involved in inhibiting the excitability of gastrointestinal muscles is pumping Ca2+ out of cells [40]. Serotonin has important regulatory functions in physiological processes such as study and memory, pain, emotion, sleep, and endocrine secretion [41]. Serotonin transporter is a membrane protein located at the presynaptic terminal, which can down-regulate 5-HT in the synaptic cleft through reuptake, antagonizing the function of serotonergic systems [42]. Studies have shown that basal 5-HT levels in patients with functional dyspepsia are down-regulated and cause abdominal and psychological symptoms through gut-brain communication. Up-regulation of SERT expression levels may be one of the mechanisms of 5-HT reduction [43, 44]. In the present study, it is shown that BHM may play a critical role in treating functional dyspepsia via regulating IL1B, TNF, TRPV1, SERT, and NOS3 signaling pathways. *In vivo* experiments have confirmed that BHM could significantly promote gastrointestinal motility, and decrease the expression of TRPV1, NOS3, and SERT. However, other genes and biological pathways may also have a particular function in the treatment of functional dyspepsia by BHM, as demonstrated in our PPI network, GO and KEGG pathway analyses, which require more in-depth study.

Using a series of predictive network model methods, this study analyzed the targets and pathways of BHM in the treatment of functional dyspepsia. Furthermore, in order to make the results of this study more reliable, we verified the main results in the underlying mechanism by *in vivo* experiments in mice. However, the research methods of network pharmacology still have some limitations. For example, the study on the chemical constituents of some herb and animal medicine is not through enough, resulting in the absence in some chemical components or targets and affecting the accuracy of study results.

## Figures and Tables

**Table 1:** The integrated targets between BHM and functional dyspepsia.

Gene names	Protein names	Degree value
IL1B	Interleukin-1 beta	31
TNF	Tumor necrosis factor	29
PTGS2	Prostaglandin G/H synthase 2	24
BDNF	Brain-derived neurotrophic factor	23
TLR4	Toll-like receptor 4	22
CXCL8	Interleukin-8	20
CASP3	Caspase-3	19
CNR1	Cannabinoid receptor 1	19
TRPV1	Transient receptor potential cation channel	19
NOS3	Nitric-oxide synthase	18
ADRB2	Beta-2 adrenergic receptor	17
GCG	Glucagon	14
COMT	Catechol O-methyltransferase	14
SLC6A4	Sodium-dependent serotonin transporter	14
PTGS1	Prostaglandin G/H synthase 1	13
NOS2	Nitric-oxide synthase	13
CCK	Cholecystokinin	13
TLR2	Toll-like receptor 2	12
TGFB1	Transforming growth factor beta-1	12
NOS1	Nitric oxide synthase	12
OPRM1	Mu-type opioid receptor	11
NOX1	NADPH oxidase 1	9
MMP3	Stromelysin-1	9
CYP2E1	Cytochrome P450 2E1	8
KIT	Mast/stem cell growth factor receptor kit	8
TACR1	Tachykinin receptor 1	8
HTR2A	5-hydroxytryptamine receptor 2A	7
ADORA1	Adenosine receptor A1	7
CYP2C19	Cytochrome P450 2C19	6
FAAH	Fatty-acid amide hydrolase 1	6
FASLG	Tumor necrosis factor ligand superfamily member 6	6
PTGER3	Prostaglandin E2 receptor EP3 subtype	6
S100A9	Protein S100-A9	5
MIF	Macrophage migration inhibitory factor	5
PYY	Peptide YY	5
F2R	Coagulation factor ii (thrombin) receptor	5
PTGER1	Prostaglandin E2 receptor EP1 subtype	5
CFTR	Cystic fibrosis transmembrane conductance regulator	5
OPRD1	Delta-type opioid receptor	5
PTGER2	Prostaglandin E2 receptor EP2 subtype	4
GHSR	Growth hormone secretagogue receptor type 1	4
ADRA2C	Alpha-2C adrenergic receptor	4
ADRA2A	Alpha-2A adrenergic receptor	4
MYLK	Myosin light chain kinase	3
CCKAR	Cholecystokinin receptor type A	3
SCN10A	Sodium channel protein type 10 subunit alpha	3
TTR	Transthyretin	2
OPRK1	Kappa-type opioid receptor	2
PTGDR	Prostaglandin D2 receptor	1
HTR4	5-hydroxytryptamine receptor 4	1
HRH3	Histamine H3 receptor	1
TP53	Cellular tumor antigen p53	0
TACR3	Neuromedin-K receptor	0
SSTR3	Somatostatin receptor type 3	0

## Data Availability

The data used to support the findings of this study are available from the corresponding author upon request.
